# Zinc Uptake by Lactic Acid Bacteria

**DOI:** 10.5402/2013/312917

**Published:** 2013-03-13

**Authors:** Alan Leonardi, Simona Zanoni, Marzia De Lucia, Alberto Amaretti, Stefano Raimondi, Maddalena Rossi

**Affiliations:** Department of Life Sciences, University of Modena and Reggio Emilia, Via Campi 183, 41100 Modena, Italy

## Abstract

The study aims to investigate zinc biosorption by strains of lactobacilli and bifidobacteria with a view to exploit them as organic matrixes for zinc dietary supplementation. Sixteen human strains of *Lactobacillus* and *Bifidobacterium* were assayed for zinc uptake. The minimum inhibitory concentration of zinc salts differed among the strains, but was never below 15 mmol L^−1^. When cultured in MRS broth containing 10 mmol L^−1^ ZnSO_4_, all the strains were capable of accumulating zinc in the range between 11 and 135 *μ*mol g^−1^. The highest amount of cell-bound zinc was obtained in *L. acidophilus* WC 0203. pH-controlled batch cultures of this strain revealed that zinc uptake started in the growth phase, but occurred mostly during the stationary phase. Pasteurized and viable cultures accumulated similar amount of zinc, suggesting that a nonmetabolically mediated mechanism is involved in zinc uptake. These results provide new perspectives on the specific use of probiotics, since *L. acidophilus* WC 0203 could function as an organic matrix for zinc incorporation. The bioavailability of *Lactobacillus*-bound zinc deserves to be investigated to provide a future basis for optimization of zinc supplementation or fortification.

## 1. Introduction

Zinc is one of the metal ions essential to life. After iron, it is the second most abundant transition metal ion in living organisms, including humans [1]. Zinc is present in all the tissues, fluids, and organs within the human body, for a total body content of approximately 1.4–2.3 g. It is necessary for catalytic, structural, and regulatory functions in hundreds of enzymes and in thousands of protein domains. Enumerating and discussing the role of zinc in these functions is far beyond the aim of this study [2–5].

The recommended dietary intake for zinc varies with age and physiological status, ranging between 5 and 18 mg day^−1^. Severe zinc deficiency causes a number of adverse physiological consequences on the epidermal, gastrointestinal, central nervous, immune, skeletal, and reproductive systems [4, 6, 7].

It has been demonstrated that the form of the trace elements affects the intake efficiency in animals. Several studies reported that certain organic compounds of trace elements (including iron, zinc, magnesium, and selenium) are more bioavailable than the inorganic forms, possibly because the mechanisms for absorption have adapted to these kinds of nutrients during species evolution [8–12]. Moreover, in order to develop biotechnological sources of trace elements for diet supplementation, microorganisms (e.g., yeast, lactobacilli, and *Spirulina* strains) were proposed as organic matrixes for incorporation of minerals [10, 13, 14]. In fact, the addition of inorganic salts into cultivation media enables the biosorption of the mineral ions by the microbial biomass. As a consequence, the biomass becomes enriched with organic forms of trace elements, which are present as complexes with amino acids, proteins, lipids, and polysaccharides [10]. The present study investigated zinc biosorption by 16 strains of lactobacilli and bifidobacteria, in the perspective to evaluate whether they can function as organic matrixes for zinc incorporation.

## 2. Materials and Methods

### 2.1. Bacterial Strains and Growth Conditions

Sixteen human strains of *Lactobacillus *and *Bifidobacterium* (Table 1) were obtained from ATCC, DSMZ, and our own collection (Deptartment of Life Sciences, University of Modena and Reggio Emilia). The strains were cultured anaerobically in MRS broth (Difco Laboratories) containing 0.5 g L^−1^ L-cysteine · HCl.

For the evaluation of zinc inhibitory concentration and metal uptake, solutions of ZnSO_4_ · 7H_2_O or ZnCl_2_ were filter sterilized (0.22 *μ*m) and added to sterile MRS-cysteine medium to obtain the appropriate concentration. All chemicals were provided by Sigma-Aldrich (Steinheim, Germany), unless otherwise specified.

### 2.2. Determination of Minimum Inhibitory Concentration

The minimum inhibitory concentration (MIC) of zinc salts was evaluated in MRS-cysteine agar plates containing ZnSO_4_ · 7H_2_O or ZnCl_2_ at the following concentrations: 0.05, 0.2, 1, 5, 10, 15, 25, 50, 75, and 100 mmol L^−1^. Overnight liquid cultures of lactobacilli or bifidobacteria were diluted with saline as appropriate; 10^4^ colony forming units were spread on the Zn-containing agar plates. Plates were inspected for growth after anaerobic incubation at 37°C for 48 h. The presence of a single colony or a faint residual haze was disregarded. The lowest concentration which inhibited bacterial growth was recorded as the MIC. 

### 2.3. Zinc Uptake

To determine zinc uptake, the strains of *Lactobacillus* and *Bifidobacterium* were cultured in MRS broth supplemented with 10 mmol L^−1^ ZnSO_4_. Zinc was quantified in biomass of 48 h cultures.

The kinetics of zinc uptake by *L. acidophilus* WC 0203 was determined in pH-controlled batch cultures. The strain was cultured in Labfors bioreactor (Infors AG, Bottmingen, Switzerland) containing 2 L of MRS-cysteine broth supplemented with 10 mmol L^−1^ ZnSO_4_. The bioreactor was inoculated (10% v/v) with an exponential-phase preculture grown in MRS-cysteine broth. The culture was kept at 37°C and was stirred at 300 rpm. The pH was continuously measured (405-DPAS probe, Mettler Toledo, Switzerland) and kept at pH 5.0 by automatic titration with 4 mol L^−1^ NaOH. Anaerobic conditions were maintained by sparging the culture with 0.05 v/v/min filter-sterilized (0.22 *μ*m) nitrogen. Samples were collected periodically to monitor growth and to quantify zinc uptake. 

**Figure 1:**
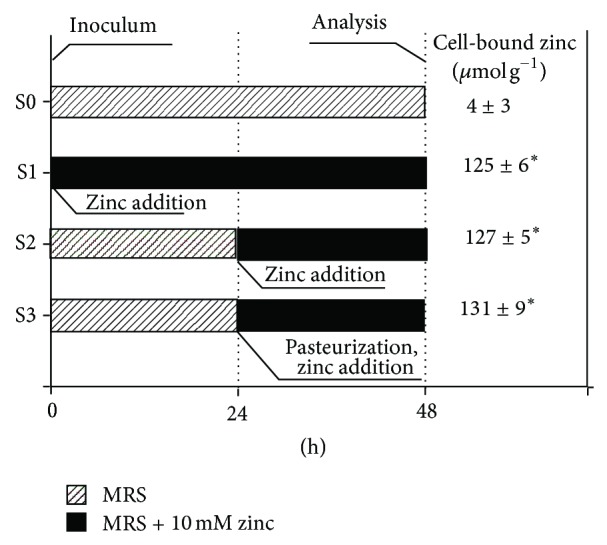
Time course of trials S0, S1, S2, and S3 and corresponding cell-bound zinc after 48 h. Dashed bar indicates incubation in MRS broth; black bar indicates incubation in MRS broth + 10 mmol L^−1^ ZnSO_4_. Values are means from three separate experiments ± SD. Means sharing a common superscript do not differ (*P* > 0.05).

To investigate the mechanism of zinc uptake by *L. acidophilus* WC 0203, pH controlled 48 h batch fermentations were carried out as follows (Figure 1): the strain was cultured in MRS-cysteine broth for 48 h cultivation in the absence (negative control) and the presence of 10 mmol L^−1^ ZnSO_4_ (S0 and S1, resp.); the strain was cultured for 24 h in MRS-cysteine broth, then the culture was supplemented with 10 mmol L^−1^ ZnSO_4_, and further incubated for 24 h (S2); the strain was cultured for 24 h in MRS-cysteine broth, then the culture was pasteurized (70°C for 15 min), supplemented with 10 mmol L^−1^ ZnSO_4_, and further incubated for 24 h (S3). For all the treatments, bacterial biomass was harvested after 48 h for zinc quantification.

### 2.4. Zinc Quantification in Bacterial Biomass

To quantify zinc in microbial biomass, the cells contained in 50 mL of culture were collected by centrifugation (6,000 ×g for 10 min at 4°C) and repeatedly washed with d.d. water until zinc concentration of in the supernatant decreased below the limit of detection (0.1 *μ*mol L^−1^).

The microbial pellet was resuspended 1 : 1.5 (w/v) with 1.44 mol L^−1^ HNO_3_ and heated in a water bath at 100°C for 40 min. The sample was centrifuged at 12,000 ×g for 5 min. The supernatant was collected and the precipitate was washed twice with 1 mL of d.d. water. The supernatant and washing waters were mixed and supplemented with 0.1 mL of 14.4 mol L^−1^ HNO_3_. The solution was brought to the volume of 5 mL with d.d. water, filtered through a 0.22 *μ*m cellulose acetate filter (Millex-GV, Millipore), and analyzed for zinc concentration using ICP-AES (Perkin Elmer Optima 4200 DV). A calibration curve was prepared using standard solutions from 0.15 to 300 *μ*mol L^−1^ Zn in 288 mmol L^−1^ HNO_3_. Cell bound zinc was expressed as *μ*mol per gram of dry biomass weight (*μ*mol g^−1^). 

### 2.5. Statistical Analysis

All values are means of three separate experiments and are expressed as mean ± standard deviation (SD). Within each strain, differences among treatments were evaluated using one-way ANOVA with repeated measures, followed by Bonferroni *post hoc* comparisons. Differences were considered statistically significant for *P* < 0.05. Differences among strains were evaluated using one-way ANOVA followed by Tukey *post hoc* comparisons. Differences were considered statistically significant for *P* < 0.05.

## 3. Results

### 3.1. Minimum Inhibitory Concentration of Zinc Salts

The minimum inhibitory concentration (MIC) of ZnCl_2_ and ZnSO_4_ was determined for 11 strains of *Lactobacillus* and 5 of *Bifidobacterium* (Table 1). The anion did not affect the MIC of zinc salts (*P* > 0.05). For all the strains of *Lactobacillus* and *Bifidobacterium*, the MIC of zinc salts was never below 15 mmol L^−1^. Among the strains of *Lactobacillus*, 6 grew abundantly on 100 mmol L^−1^ zinc (*L*.* acidophilus *WC 0202,* L*.* acidophilus *DSMZ 20552,* L*.* brevis *ATCC 4006,* L*.* gasseri *WC 0213,* L*.* plantarum *WC 0214, and* L*.* reuteri *WC 0215). Among the strains of* Bifidobacterium*, the highest MIC of zinc (100 mmol L^−1^) was observed for *B. breve* WC 0480.

### 3.2. Zinc Uptake

Cell-bound zinc was determined after 48 h of growth in MRS versus MRS supplemented with 10 mmol L^−1^ zinc. Zinc concentration in the un-supplemented MRS broth was determined and was 0.015 mmol L^−1^. In this medium, all the strains accumulated less than 4.6 *μ*mol g^−1^. Cell-bound zinc was always significantly higher (*P* < 0.05) when the strains were cultured in zinc-supplemented MRS, even if wide differences were observed among the strains (Table 1). In the strains of *Bifidobacterium*, zinc uptake ranged between 15 and 32 *μ*mol g^−1^. In the strains of *Lactobacillus*, zinc ranged between 11 and 135 *μ*mol g^−1^ and was the highest in *L. acidophilus* WC 0203. 

### 3.3. Kinetics and Mechanism of Zinc Uptake by *Lactobacillus Acidophilus* WC 0203

**Figure 2:**
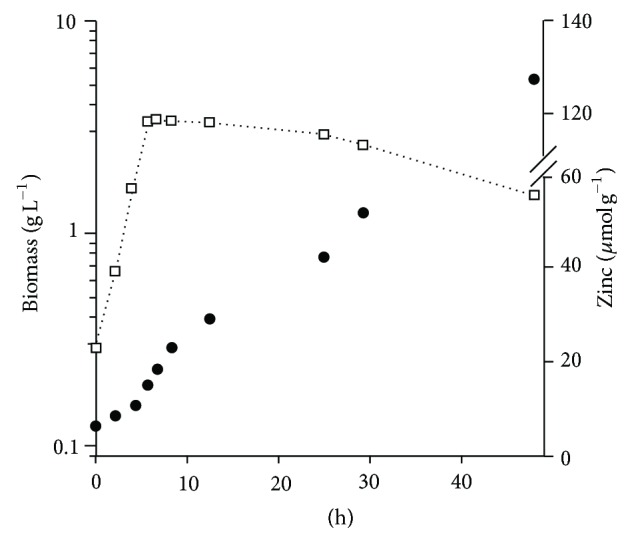
Batch culture of *Lactobacillus acidophilus* WC 0203 in MRS broth containing 10 mmol L^−1^. Symbols: □, biomass dry weight; •, cell-bound zinc.

The relationship between microbial growth and zinc uptake was investigated in* L. acidophilus *WC 0203 using pH controlled batch fermentations in zinc-supplemented MRS (Figure 2). The strain grew with a specific growth rate *μ* of 0.39 h^−1^. After 6 h glucose was depleted (data not shown) and the culture entered into the stationary phase, giving a biomass/glucose yield of 0.037. Zinc uptake occurred during both the exponential and the stationary phases. Cell-bound zinc was 20 *μ*mol g^−1^ at the end of the exponential growth. Zinc uptake continued throughout the stationary phase up to 134 *μ*mol g^−1^ after 48 h of incubation (Figure 2). 

In order to explore the mechanism of zinc uptake by* L. acidophilus *WC 0203, cell-bound zinc was measured after 48 h of cultivation in 4 different trials (Figure 1). When the strain was cultured without Zn supplementation (S0), cell-bound zinc was 4 *μ*mol g^−1^. When zinc was added at the inoculum (S1), to stationary cultures of 24 h (S2), or to pasteurized cultures of 24 h (S3), cell-bound zinc was 127, 125, and 131 *μ*mol g^−1^, respectively. Zinc level in S1, S2, and S3 trials was significantly higher (*P* < 0.05) than in S0; differences among S1, S2, and S3 were not statistically significant (*P* > 0.05).

## 4. Discussion

Some species of* Lactobacillus* and *Bifidobacterium* are extensively used as probiotics in fermented foods and nutraceutical products. Among probiotics beneficial effects, lactobacilli and bifidobacteria acidify the large intestine restricting putrefactive and potentially pathogenic bacteria, cause immune-stimulation, exert antioxidant and anticarcinogenic activities, produce vitamins, and activate biologically active compounds [15–18]. An increasing number of complete genome sequences of lactic acid bacteria are being added to databases and plenty of information about physiology, metabolism, and probiotic properties is available. However, little is known about the ability of these bacteria to bind metals. Only recently few strains of lactobacilli have been investigated for zinc biosorption [19]. In order to propose probiotic carriers of organic zinc, the present study investigated the ability of 16 human strains of *Lactobacillus* and *Bifidobacterium* to bind zinc.

Results from our screen revealed a significant variation in both MIC and metal uptake of zinc among the various strains tested. Cell-bound zinc ranged between 15 and 32 *μ*mol g^−1^ in bifidobacteria and between 11 and 135 *μ*mol g^−1^ in lactobacilli. Any correlation between MIC and cell-bound zinc seems to be excluded (*R* ^2^ = 0.064). The strains which presented both the highest and the lowest level of zinc uptake belonged to the species *L. acidophilus*. *L. acidophilus* WC 0203 bound 135 *μ*mol g^−1^; *L. acidophilus* DSMZ 20552 bound 11 *μ*mol g^−1^; the corresponding MICs were 15 mmol L^−1^ and higher than 100 mmol L^−1^, respectively.

A deeper characterization of zinc uptake mechanism and kinetics of *L. acidophilus* WC 0203 revealed that the amount of cell-bound zinc increased progressively during a batch process. However, most of the uptake occurred during the stationary phase, when growth ceased. In fact, zinc content was similar in biomass collected from 48 h batch cultures where the salt was added at the beginning of the process and in biomass of cultures where zinc was supplied in the late stationary phase. A passive mechanism is probably involved in zinc uptake by *L. acidophilus* WC 0203, because pasteurized and viable cultures accumulated similar amount of zinc. Under these conditions, biomass incorporated up to 131 *μ*mol g^−1^ zinc, corresponding to approximately 8.5 mg g^−1^. Since zinc uptake seems to occur through nonmetabolically mediated mechanism of uptake, it is conceivable that zinc incorporation could be further enhanced if stationary phase cells are exposed to zinc concentrations even higher than the MIC. Our results are in agreement with the observation that, in *Lactobacillus mesenteroides*, zinc was mostly bound to carboxylate groups of proteins and that the cell wall was the major site of zinc localization, even though active internalization progressively occurred too [19].

Diets composed primarily of plant ingredients contain diverse antinutritional factors which inhibit mineral absorption. Among them, phytate exerts the major inhibitory effects on zinc absorption. The phosphate groups of phytate can form strong and insoluble complexes with zinc ions, causing them to escape absorption and to be excreted with feces. Low molecular weight ligands of zinc can form soluble complexes with zinc, thus counteracting the effect of phytate. Therefore, chelators (e.g., EDTA), amino acids (e.g., glycine, histidine, and methionine), and organic acids (e.g., citrate) have been used to enhance zinc bioavailability [20, 21]. Certain animal proteins of food were demonstrated to improve zinc absorption as well, likely due to the amino acids and peptides, which are released with digestion, that keep zinc in solution [22]. 

Similar to zinc, the organic forms of other trace elements (e.g., magnesium and iron) are more bioavailable and tend to be more effective against deficiency than the mineral ones [8–12]. For the same reason, the utilization of microbial biomass for the delivery of microelements has been increasingly explored. Above all, the dietary supplementation with selenium-enriched biomass of yeast or lactobacilli resulted in higher concentrations of selenium in tissues and body fluids than supplementation with inorganic selenium did [13, 14]. 

The ability of *L. acidophilus* WC 0203 to function as an organic matrix for zinc incorporation could be exploited to supply organic forms of this metal. This approach for zinc supplementation could be of interest if zinc complexes are released from enriched biomass upon passing through the upper gastrointestinal tract, counteracting the effects of antinutritional factors. If biosorbed zinc is fully bioavailable, 1 g of zinc-enriched biomass of *L. acidophilus* WC 0203 could provide the host with a zinc amount (8.5 mg) staying within the recommended daily intake (5–18 mg). Therefore, the bioavailability of *Lactobacillus*-bound zinc may deserve deeper investigation.

## 5. Conclusions

The results of this study provide new perspectives on the specific use of probiotics, such as to combine the healthy probiotic effects of the genus *Lactobacillus* with the delivery of organic zinc, preventing sequestration by antinutritional factors.

## Figures and Tables

**Table 1:** Minimum inhibitory concentration (MIC) of zinc salts and cell bound zinc concentration in *Lactobacillus* and *Bifidobacterium* strains.

Strain	MIC∗ (mmol L^−1^)	Cell-bound Zn^†^ (*μ*mol g^−1^)
*Lactobacillus acidophilus* ATCC 4356	15	107
*Lactobacillus acidophilus* DSMZ 20552	>100	11^d^
*Lactobacillus acidophilus* WC 0202	>100	63^a^
*Lactobacillus acidophilus* WC 0203	15	135
*Lactobacillus brevis* ATCC 4006	>100	86
*Lactobacillus buchneri* WC 0204	50	50^b^
*Lactobacillus casei* WC 0205	15	29^c^
*Lactobacillus gasseri* WC 0213	>100	21^c,d^
*Lactobacillus parabuchneri* WC 0283	100	18
*Lactobacillus plantarum* WC 0214	>100	50^b^
*Lactobacillus reuteri* WC 0215	>100	61^a,b^
*Bifidobacterium breve* WC 0421	15	32^a^
*Bifidobacterium breve* WC 0480	100	15^b^
*Bifidobacterium breve* WC 0481	50	17^b^
*Bifidobacterium infantis* WC 0460	15	32^a^
*Bifidobacterium pseudocatenulatum* WC 0455	15	31^a^

^*^MIC was determined in MRS agar plates containing ZnCl_2_ or ZnSO_4_. Difference between ZnCl_2_ and ZnSO_4 _was not significant (*P* > 0.05).

^†^Zinc uptake was determined in MRS supplemented with 10 mmol L^−1^ ZnSO_4_. Values are means from three separate experiments, standard deviation was less than 5**** *μ*mol g^−1^. Within a microbial genus, means sharing a common superscript do not differ (*P* > 0.05).
